# Factors Associated with High Live Release for Dogs at a Large, Open-Admission, Municipal Shelter

**DOI:** 10.3390/ani8040045

**Published:** 2018-03-28

**Authors:** Gary J. Patronek, Abbi Crowe

**Affiliations:** 1 Center for Animals and Public Policy, Cummings School of Veterinary Medicine, Tufts University, North Grafton, MA 01536, USA; 2 Best Friends Animal Society, Kanab, UT 84741, USA; abbic@bestfriends.org

**Keywords:** adoption, animal shelter, dogs, foster programs, length of stay, live release, outcomes, rehoming

## Abstract

**Simple Summary:**

Better understanding of factors contributing to live release (rehoming) may help shelters improve outcomes. In this study, data were analyzed for all dogs (n = 21,409) admitted over a two-year period for the primary purpose of rehoming at a high-volume, open-intake municipal shelter performing animal control (the Pima Animal Care Center in Tucson, Arizona). Results show that >88% of eligible dogs were rehomed through adoption to the public or transfer to a rescue group. Temporary placement into interim foster homes of dogs who were either not immediately eligible or not strong candidates for adoption due to reasons such as age or health, increased the odds of live release after subsequent return to the shelter, especially for adult dogs. Dogs returned to the shelter after unsuccessful adoption had a live-release advantage as well, which suggested that the temporary experience in a home was not detrimental and may have facilitated the likelihood of live release after return, akin to a foster situation. Over a fifth (21.1%) of dogs originally brought to the shelter for owner-requested euthanasia were determined to be potentially savable and ultimately rehomed. It remains to be determined whether other large, open intake shelters performing animal control can replicate these results.

**Abstract:**

Better understanding of factors contributing to live release (rehoming) may help shelters improve outcomes. In this cross-sectional, exploratory, non-interventional study, data for all intakes (n = 21,409) for dogs eligible for rehoming from 1 January 2015 to 31 December 2016 are analyzed to identify such factors. Live release was >88%. A total of 1510 (7.1%) dogs interacted with the foster care system, 98.9% of whom had live release. Foster care increased the odds of live release by about five-fold for all dogs (odds ratio (OR) 5.30 (95% confidence interval (CI): 3.13; 8.97), p < 0.001) and by >20-fold for adult dogs (OR 22.2 (95% CI: 5.48; 90.2), p < 0.001) compared to first-time owner-surrendered dogs. Dogs returned from foster care had a 70% reduction in health concerns, as judged by intake staff, compared with dogs sent to foster. In addition to saving 2882 lives, the rescue network utilized by this shelter was estimated as having reduced in-shelter care needs by 13,409 animal care-days over two years. Dogs returned from adoption also had increased odds of live release (OR 4.74 (95% CI: 3.02; 7.44), p < 0.0001). Nearly a third (29.6%) of dogs originally brought in by owners for euthanasia were determined to be potentially savable, and a fifth of the original group (21.1%) were ultimately placed. Less than 4% of dogs presented with behavioral concerns at intake. It remains to be determined whether other large, open intake shelters performing animal control can replicate these results.

## 1. Introduction

Much of the literature dealing with the epidemiology of animal shelter populations has explored basic descriptive statistics [1,2,3,4,5,6] and/or reasons for relinquishment [7,8,9,10,11,12], with the hope that better understanding of these factors could lead to interventions that would reduce intake, and therefore ultimately decrease the euthanasia of homeless pets. According to one database, live release (i.e., rehoming vs. euthanasia or natural death) for dogs after admission to shelters across the US is reported to be quite variable [13], but factors contributing to these differences, including return to owner and transfer to rescue, have received limited attention. For example, in 1998, Posage et al. [14] examined owner-completed intake questionnaires from 1468 dogs relinquished at a single open-admission shelter serving an urban population in Ohio. They reported that small size, being purebred, male sex, having lighter coat colors, and absence of health problems were significant predictors of adoption, but in a multivariate model, the factors they identified explained less than 5% of the variance in survival. Their analysis also excluded “pit-bull”-type dogs, as those dogs were not made available for adoption at the time. Similarly, Lepper et al. [15] have reported that, in a California animal shelter, younger age, female sex, and purebred status were associated with increased likelihood of adoption vs. euthanasia.

Sinski et al. [16] focused on determining whether having a black coat color (the “black dog bias syndrome”) was associated with the likelihood of adoption vs. euthanasia for dogs in a single public shelter in a large metropolitan area (Louisville, KY, USA), but also reported data for other risk factors. Briefly, their univariate analysis indicated that black-colored dogs were 23.8% less likely to be adopted than partially black or non-black dogs. However, this relationship was also found to have complex interactions with size and breed, so the results remained inconclusive. They also reported that “pit bull”-type dogs were 81.4% less likely to be adopted than Labrador Retrievers. Sex of the dogs was not related to outcome. Svoboda and Hoffman [17] also explored the veracity of this putative “black dog bias syndrome” in two animal shelters in the US Pacific Northwest, one of which was open admission and the other limited admission. Using a univariate analysis, they reported that there was no difference in either length of availability for adoption or live release for black dogs. Older dogs and “bully breeds” were reported to have longer length of availability before adoption, as well as poorer outcomes than younger dogs or dogs of other breeds. At one of the shelters, males were also less likely than females to have a live release. Brown et al. [18] evaluated the effects of phenotypic characteristics on length of stay at two “No-Kill” animal shelters in rural New York State, and reported that length of stay was shortest for puppies and increased linearly with age. In that study, neither coat color nor sex influenced length of stay. For adult dogs, length of stay was longest for dogs identified as members of the “Bully” group and “Guard” group, while dogs identified as “giant” had the shortest length of stay. 

Few of these risk factor studies have examined both live release and length of stay, and, to our knowledge, even when individual risk factors were adjusted for potential confounders using a multivariate analysis, no study has examined the effect of multiple risk factors acting in combination on overall probability of live release. We have not identified any published data on the effect of foster care on live release. Although numerous studies have documented adoption return to shelters, there is little information about the fate of those returned dogs once back in the shelter.

Today, more and more shelters and communities are working towards the goal of saving every placeable dog. The shelters believed to face the greatest challenges in achieving this goal are high-volume, municipal, open-admission facilities that are responsible for animal control services. Lower live release may be assumed as inevitable in such shelters due to their high volume, unselected population of dogs, unpredictable timing of intakes, and limited resources to devote to placement and adoption, compared to limited admission shelters (i.e., those that do not euthanize for space, that limit their intake volume and/or that remain selective as to eligibility for intake with respect to characteristics such as age, breed, size, health, or behavior). In this cross-sectional study, our aim was to identify, for dogs admitted for rehoming, factors associated with live release and shorter length of stay from one such open-admission shelter that challenges this stereotype.

## 2. Materials and Methods

### 2.1. Source of Data and Shelter Characteristics

The Pima Animal Care Center (PACC) in Tucson, Arizona is an open-admissions shelter taking in approximately 19,000 pets each year from Pima County, which covers more than 9000 square miles (about the size of the US state of New Hampshire) and had an estimated population of 1.01 million during the time period of the study [19]. About one-third of the intakes are cats and two-thirds dogs, with approximately 60% of the dogs being strays. This amounts to an annual per-capita intake of about 12.5 dogs and 6.3 cats per 1000 persons. The poverty rate is estimated at 18.2%. During the time of the study, PACC was a department under the Health Services division of the county, and the budget of over $9 million was part of the overall Health Services fund. The annual budget was supplemented by donations from a non-profit “Friends of the Shelter” group to support programs such as veterinary medical care.

PACC is the only entity performing animal control for the area, and stray dogs are typically held for three days to allow for return to owner. During the period of the study, the shelter employed approximately 95 staff members (~60 animal care, medical and support staff; 25 animal control officers/humane law enforcement; and 10 executive/management). Approximately 18 of the animal care staff were contracted workers or were assigned from a correctional institution. PACC also has relationships with approximately 1500 volunteers and foster caregivers, and over 100 rescue partners, to assist with rehoming efforts. 

### 2.2. Records and Variables

A total of 29,213 canine records were downloaded from the shelter’s computerized database (Chameleon/CMS software, version 44J, HLP Inc., Littleton, CO, USA). There were 7804 records (26.7%) excluded from the analysis (Table 1). To focus the analysis on dogs whose length of stay and/or availability for adoption was not unduly influenced by factors outside of the shelter’s control, we excluded records for dogs whose primary reason for intake was not rehoming (i.e., dogs admitted for rabies quarantine, dogs confiscated by law enforcement, and dogs brought in by owners for euthanasia and subsequently euthanized). The final analysis set consisted of 21,409 dog intake records. Variables in the original dataset included sex, estimated age, weight, sterilization status, estimated size, intake date, outcome date, intake type (i.e., owner surrender, stray, return from adoption, and return from foster care), outcome type (i.e., adoption, sent to foster care, transferred to rescue, died, and euthanized), and primary color. Foster care was defined as placement in an interim home in the community for purposes of medical or behavioral rehabilitation prior to being returned to the shelter for permanent adoption. A “rescue” was defined as an individual, an informal group, or formal (e.g., recognized non-profit organization) organization that would accept the dog and assume responsibility for placement into a permanent home instead of being returned to PACC. 

With respect to other variables in the database, staff were required by the computer system to make an entry for primary breed, and, if desired, secondary breed (which could include the designation of “Mix”) at intake. Breed designations may have been provided by owners, by another person bringing the dog into the shelter, or be staff opinion based on the dog’s appearance. It was not possible to independently confirm accuracy of these attributions, as pedigrees, DNA analyses, and other reliable information about parentage were not available. 

### 2.3. Data Analysis

All data were maintained in an electronic spreadsheet. Descriptive statistics were tabulated and absolute differences in live release for different characteristics were calculated and presented as proportions. The relative differences in live release for individual traits vs. a comparator category were calculated using logistic regression. Logistic regression was also used to calculate the odds of length of stay ≤7 days vs. >7 days. Wizard^TM^ software (version 1.9.12, http://www.wizardmac.com/) was used to run the logistic models. A linear model using the calculated regression coefficients was used to predict the probability of live release for various combinations of traits (risk factors).

For purposes of simplifying the analysis, some variables were recoded. Estimated age was used to assign dogs to the following five categories: puppy, ≤6 months; juvenile, 6–12 months; young adult, more than 1 to less than 3 years; adult, from 3 to less than 8 years; and older adult, ≥8 years. Condition at intake was further categorized into just three groups: no concerns (listed as “normal” at intake), minor concerns (i.e., minor health problems, manageable behavior problems or old age), and major concerns (i.e., severe health problems, severe behavior problems or aggression). This was a subjective assessment made by intake staff based on their experience and own assessment, without specific criteria or policy.

With respect to the mandatory entries about breed in the database, other work has shown that different observers often disagree on breed makeup, and that breed designations may not correlate with DNA analyses [20,21,22,23]. Due to the uncertainty about breed designations and the large number of possible combinations of primary and secondary breeds in the database, we did not attempt to perform any analyses based on individual breed designations. To avoid promulgating data we knew were likely to be unreliable, and recognizing that one common denominator in these designations by staff was dogs’ physical appearance, we created a single variable called “Blockhead-type appearance” in which we placed all dogs whose primary or secondary breed designation was potentially consistent with the appearance of dogs of a stigmatized type or breed (i.e., American Staffordshire Terrier, American Pit Bull Terrier, Boxer, American Bulldog, Bull Mastiff, Canine Corso, Dogo Argentino, English Bulldog, Mastiff, “pit bull”-type dog, and Rottweiler), who may be more difficult to place after being admitted to a shelter [2,15,24,25,26,27,28]. For all of the reasons listed above, it was not possible to consistently and reliably determine if dogs were purebred or mixed breed. Therefore, we only examined whether one or >1 breed was listed in the database for a given dog. 

The variables “size” and “weight” were often missing and deemed unreliable in the dataset and were discarded. To roughly approximate adult size, the likely adult weight of the primary breed the dog was listed as resembling based on American Kennel Club (AKC) average weights [29] was used to place dogs into the categories of “small”, “medium”, “large”, and “giant”. Sterilization status was not included in the analysis because, by statute, the shelter was required to spay or neuter all dogs prior to adoption, and sterilization status on intake was not recorded. Thus, it was not possible to determine which dogs were already sterilized at intake and which were sterilized prior to release from the shelter. 

## 3. Results

### 3.1. Overall Live Release and Length of Stay

The final dataset of 21,409 dog intakes included records for 18,846 individual dogs considered potential candidates for rehoming, with 2563 (12%) records representing another intake for the same dog (n = 3760, two intakes; n = 783, three intakes; n = 156, four intakes; and n = 55, five intakes). Using data for 21,409 dog intakes, overall live release (as percent of intakes) of dogs deemed potential candidates for rehoming was calculated as 88.6% (Table 2). Using only last intake, live release was 16,398/18,846 (87%). The demographic characteristics were as follows for dogs with single vs. multiple intakes, respectively: female, 47.6% vs. 49.7%; single breed designation, 36.3% vs. 30.8%; blockhead appearance, 26.9% vs. 33%; puppies, 20.9% vs. 24.5%; and older adults, 6% vs. 4%. 

**Figure 1:**
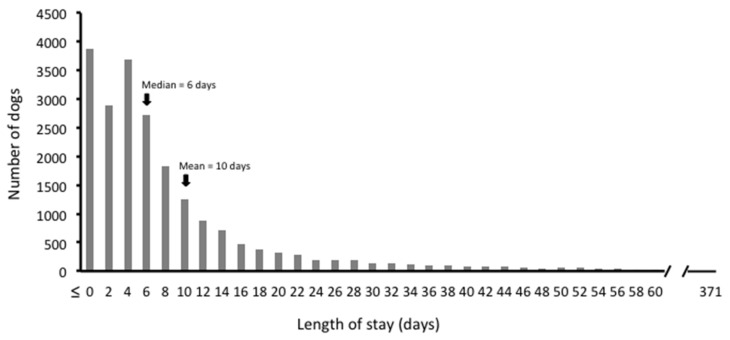
Distribution of length of stay for 21,409 dogs admitted for rehoming at Pima County Care Center, 2015–2016.

Length of stay (LOS) was skewed to the right (Figure 1), with a mean ± standard deviation LOS of 10 ± 14.5 days and a median LOS of six days (range, 0–371 days). The effect of individual dog characteristics on live release and LOS is explored below.

### 3.2. Age

With respect to absolute differences, live release was highest for puppies (92.2%) and juveniles (91.8%), followed by young adults (90.1%), adults (89.2%), and older adults (84.7%). Estimated age was not available for 2842 (13.3%) dogs, and those dogs had a live release rate of 76.4%. In terms of relative differences, in both univariate and multivariate analysis, the oldest age group had about half the odds of live release as a puppy (Table S1). The odds of release within a week (LOS ≤ 7 days) of intake also decreased progressively with increasing age.

### 3.3. Sex

Live release rate for 11,038 male dogs was 87.4% compared to 90.5% for 10,181 females. Male dogs had a significantly lower odds of live release compared to female dogs (odds ratio (OR) 0.73 (95% confidence interval (CI): 0.66; 0.79), p < 0.001 and adjustment for other factors in a multivariate analysis (Table S1) had little effect (OR 0.68 (95% CI: 0.61; 0.76), p < 0.001); however, males were not less likely to be rehomed within seven days than females (OR 0.98 (95% CI: 0.92; 1.04), p = 0.46). 

### 3.4. Intake Type

Among intake categories, owner surrendered dogs and stray dogs had absolute live release of 88.6% and 87.4%, respectively (Table 3). When stray dogs returned to owners (RTO) were excluded from the analysis (n = 2439, 19.1%), the live release via adoption or rescue for the remaining 9911 dogs was 83.7%, which is only slightly lower than that for strays overall, including RTO (87.4%). Importantly, dogs returned to the shelter after a period in foster care or after an unsuccessful adoption (<30 days) were nearly all released alive (98.1% and 96.9%, respectively). It should be noted that adoption returns ≥30 days were entered into the database as owner surrenders, and thus not able to be tracked separately. In terms of relative differences, in the univariate analysis, stray dogs were less likely to have a live release outcome than owner-surrendered dogs (OR 0.90 (95% CI: 0.82; 0.98), p = 0.016), which became worse when strays reclaimed by their owners were removed from the analysis (OR 0.73 (95% CI: 0.67; 0.80), p < 0.001). However, after adjustment for other factors (age, sex, condition at intake, color, estimated size, number of breeds listed, and “blockhead-type” appearance) stray dogs were more likely to have a live release outcome (OR, 1.25 (95% CI: 1.11; 1.39), p < 0.001), although that advantage disappeared if owner-reclaimed stray dogs were removed from the analysis (OR 1.01 (95% CI: 0.90; 1.13), p = 0.83) (Table S1). 

Dogs brought in by owners for euthanasia (n = 2631) were originally excluded from analysis, since it was assumed they were not eligible for rehoming. However, upon inspection of the data, it was found that 780 (29.6%) of those dogs were converted to owner surrender for possible placement after discussion during intake with owners. The majority of those dogs (557/780; 71.4%), representing over one-fifth (21.2%) of all dogs brought in for owner-requested euthanasia, were eventually placed through adoption (n = 293), rescue (n = 219), or temporarily rehomed via interim foster care (n = 45). 

**Figure 2:**
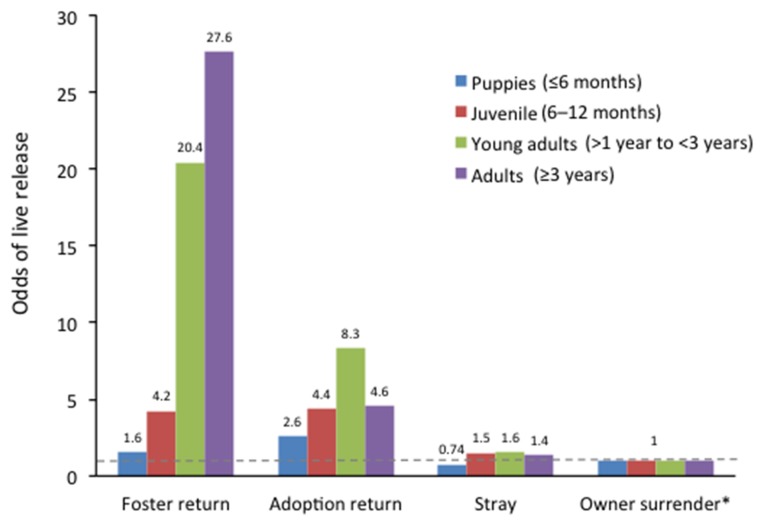
Relationship between intake type and odds of live release for dog intakes at Pima Animal Care Center, by estimated or reported age. * Owner surrender is reference category for each age group, odds ratio = 1. Odds ratios were not adjusted for factors other than age.

Dogs returned from adoption from PACC had a four- to five-fold higher odds of live release compared to first-time owner surrendered dogs (original source of dog unknown) to PACC even after adjustment for other factors (OR 4.74 (95% CI: 3.02; 7.44), p < 0.001). Similarly, dogs returned to the shelter after interim residence in a foster home were more than five times more likely to have a live release (OR 5.30 (95% CI: 3.13; 8.97), p < 0.001) than an owner-surrendered dog. When puppies sent to foster were excluded, the effect for adult dogs was even more marked, with dogs returned from foster having over a 20-fold increase in odds of live release (OR 22.2 (95% CI: 5.48; 90.2), p < 0.001) compared to owner-surrendered dogs (Figure 2; Table S1). 

There were 856 dogs who experienced foster care, with almost all being sent to foster care for medical reasons. When going out to foster care, dogs had a much higher prevalence of concerns (20.3% major and 28.4% minor vs. 51.3% no concerns) compared with the same dogs returning from foster (5.5% major and 9.1% minor vs. 85.1% no concerns). This amounted to a reduction of 70% in the prevalence of major or minor concerns compared with dogs going out to foster. By comparison, single-intake dogs who were adopted without going through foster had a prevalence of 4.3% major and 10% minor concerns vs. 85.7% no concerns.

With respect to permanent transfer to rescue, one way to gauge the potential impact of a robust rescue network, beyond facilitating rehabilitation and/or live release, is to assess the effect on shelter capacity. Overall, 2882 dogs were permanently transferred to a rescue partner. These dogs spent a total of 23,552 animal care-days in the shelter prior to transfer (mean of 8.2 days). Had they spent instead the mean number of days in the shelter as for adopted dogs (12.7 days), the total number of animal care-days in residence would have been 36,601, implying a difference of 13,409 fewer animal care-days as a result of the transfer. 

### 3.5. Medical and Behavioral Health at Intake

Dogs deemed “normal” at intake (i.e., having no health or behavioral concerns and not being aged, n = 16,699) had a live release of 92.7%, whereas dogs with minor concerns (n = 2887) had a live release of 83.7%, and dogs with major concerns (n = 1823) had a live release of 57.8%. Of that latter group coded as having major concerns at intake, the vast majority were health-related (n = 1465, 80.4%). Indeed, dogs deemed to have potentially major behavior concerns, including aggression, at intake (n = 331) represented only 1.5% of all dogs admitted for placement. An additional 464 (2.2%) of dogs were deemed to have manageable behavior problems at intake. The total proportion of dogs presenting with behavior concerns at intake was 3.7%.

Comparatively speaking, there was a statistically significant trend (χ^2^ = 2056, p < 0.0001) for health or medical concerns of increasing severity at intake to be associated with a progressively decreased odds of live release (OR 0.44 (95% CI: 0.38; 0.50), p < 0.001 and OR 0.13 (95% CI: 0.12; 0.15), p < 0.0001 for minor and major concerns, respectively, compared to dogs with no concerns). This factor seemed to be independent of the presence of other factors, as there was very little change in the odds ratios for minor or major concerns in the multivariate analysis (Table S1). 

### 3.6. Appearance/Morphology

With respect to color, the live release for black and non-black dogs was identical at 88.5% and being black had no effect on odds of live release (Table S1). As far as estimated size, small dogs had the highest live release (93.9%). Otherwise, there was no clear association between estimated adult size and probability of live release (medium, 83.5%; large, 89.1%; and giant, 85.3%). Compared to being estimated as having a small size as an adult, dogs of all other estimated sizes had significantly lower odds of live release, with no obvious trend once a dog was no longer small (Table S1).

Dogs identified with only a single breed designation in the database (n = 7511) had a live release of 87.6%, whereas dogs with both a primary and secondary breed listed (n = 13,893) had a live release of 89.1%. With respect to relative differences in live release, having >1 breed listed was associated with a small, but significantly increased odds of live release in the univariate analysis (OR 1.16 (95% CI: 1.06; 1.26), p < 0.001), but that difference disappeared after controlling for other factors (OR 0.96 (95% CI: 0.86; 1.08), p = 0.49). Those dogs were also more likely to be placed within one week of intake (Table S1). 

Dogs whose primary or secondary breed designation was consistent with the appearance of dogs stigmatized due to their presumed breed (“Blockhead-type” dogs) represented over a quarter of all dogs (n = 6078, 28.4%), with a live release of 80.5% compared with 91.7% for 15,325 dogs not in that category. Those dogs also had a significantly lower odd of live release compared to other dogs, both in the univariate analysis (OR 0.37 (95% CI: 0.34; 0.41), p < 0.001, and after adjustment for other factors in the multivariate analysis (OR 0.46 (95% CI: 0.41; 0.52); p < 0.001) (Table S1). They were also less likely to be placed within a week (OR 0.72 (95% CI: 0.67; 0.77), p < 0.001.

### 3.7. Effect of Factors in Combination 

**Figure 3:**
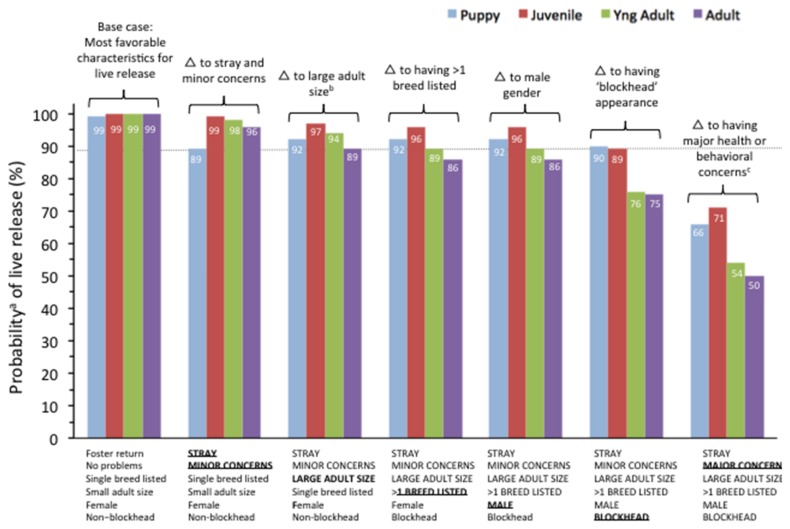
Probability of live release for dog intakes at Pima Animal Care Center according to different combinations of characteristics ^b^, by estimated or reported age category. Δ = change characteristic. Characteristics changed from previous column are shown in bold and underlined; dotted line shows overall, average live release rate across all groups. ^a^ From linear model with regression coefficients for each characteristic adjusted for age. ^b^ Size is approximated in broad categories (small, medium, and large) from primary breed attribution in Chameleon database using published breed standards for weight. ^c^ Most (98.5%) were medical/health concerns rather than behavioral.

Identifying statistically significant risk factors, even after adjustment for other factors, is of limited utility because risk factors operate collectively to influence live release for individual dogs. The probability of live release according to various combinations of dog characteristics is shown in Figure 3. The most favorable combination of characteristics, representing near-certainty of live release, was a small, female dog without a “blockhead”-type appearance, with no medical or behavioral concerns and with only a single breed designated. When the panel of characteristics was changed to a stray dog with minor behavioral or medical concerns, only puppies experienced adverse odds of live release (reduction of about 10%). Changing to large adult size, having >1 breed listed, or male sex had at most a modest effect on reducing the probability of live release (3–10%, depending on age), with the most marked effect being on adult dogs. Possibly having a “blockhead-type” appearance reduced the probability of live release, but this effect, in conjunction with the previous combinations of characteristics, was most evident for adult dogs (~75% probability of live release) compared with puppies and juvenile dogs (~90% probability of live release) (Figure 3). Adding major health or behavioral concerns to this profile also had an adverse effect on reducing the probability of live release, again particularly for adult dogs (~50% probability of live release). 

## 4. Discussion

In this high-volume, open-admission shelter that also performed animal control functions for a human population of about one million, overall live release for dogs whose primary reason for intake was rehoming approached 90%, and for dogs with the most optimal combination of characteristics, was a near certainty. This appears to be a marked improvement over historical trends [30], as have been described in detail for the Denver metropolitan area as one example [6]. Unfortunately, nationally representative statistics from animal shelters are not available, and data from convenience samples, even large ones, may include statistics from shelters that are not directly comparable due to being smaller in size, limited admission, dog-only, cat-only, not performing animal control, or otherwise systematically different. To attempt to put the live release from PACC in perspective, data from five other shelters with similar levels of intake of dogs and cats and open-admissions policies who voluntarily report data to the Shelter Animals Account collaborative project were reviewed: the net live release for dogs were 32.7%, 41.3%, 64.7%, 76.2%, and 86.2% [13].

To our knowledge, this is the first study to report the joint influence of multiple risk factors on probability of live release from a shelter. This allows assessment of the actual impact of even a large and statistically significant risk factor on live release once the contribution of other factors is taken into account. An important take-away from these data is that, despite certain characteristics being more favorable than others, there was no specific combination of age, size, appearance, sex, source of the dog, or condition at intake that automatically precluded placement. Our results are consistent with those of Garrison and Weiss [26] indicating that adoption decisions are complex and influenced by a multitude of factors, and that no single attribute may drive choice. Comparing our results with other studies reporting odds ratios is problematic due use of different reference categories within variables or even categories from unrelated variables in other studies. Nevertheless, our results are also consistent with other work demonstrating the importance of appearance on visitor behavior [31] and likelihood of adoption [32]. 

Individually, several other findings were mostly as expected. For example, size had an important effect on live release—once dogs were no longer “small”, their odds of live release were reduced, with dogs estimated to fall in the range of “giant” having the lowest odds (adjusted OR 0.57 (95% CI: 0.42; 0.76)) compared with small dogs. In addition, as expected, being a puppy had the highest odds of live release, but, beyond that age, there was no clear trend for increasing age to be associated with decreased probability of live release. However, if other risk factors were present, they tended to affect live release for older dogs more than younger dogs (Figure 3). Similar trends were observed for size, with the effect of being larger tending to have the most impact on older dogs (data not shown). Male dogs also had significantly reduced odds of live release compared to female dogs (OR 0.68 (95% CI: 0.61; 0.76), p < 0.001), although the absolute difference was small (87.4% vs. 90.5%, for males vs. females, respectively. Despite their reduced odds of live release, males were not less likely to be placed within seven days, a comparison which held after adjustment for other factors (Table S1). 

Utilization of temporary foster care had a marked effect on improving odds of live release at this shelter. Although various types of foster care have been used by animal shelters for many years, these programs are largely ad-hoc efforts that have not been systematically evaluated, standardized or formalized. One paper has noted the feasibility of having foster homes be responsible for canine adoptions, a program, which merges aspects of foster and rescue [33], but, otherwise, to our knowledge, formal evaluations have not been published. Much work remains to be done to determine optimal practices for foster programs, the extent to which foster programs can be expanded, how/if they should be financially supported, and how to ensure an appropriate standard of care and safety is maintained for dogs once away from day-to-day supervision by shelter staff. Details of the foster care program at PACC are provided in Appendix A.

Two surprising findings emerged from this exploratory, non-interventional study of shelter records. The first was that dogs returned from adoption (n = 816) were almost all subsequently re-adopted (n = 695, 85.2%, median LOS four days) or placed in interim foster care or transferred to a rescue group (n = 76, 9.3%). Adoption returns are considered quite pejoratively in the shelter community (the so-called “failed adoption” [14,34,35]), but, in this study, at least based on live release outcomes, there was no evidence that dogs were adversely affected by the experience of being temporarily rehomed. Indeed, the experience may have been more akin to a foster situation, in that the dogs were removed to a home environment and there was an opportunity to gather updated information about the dog’s real behavior and needs. In fact, data suggest this experience appears to have facilitated a subsequent adoption, the latter more likely to occur within one week of return (OR 1.44 (95% CI: 1.22; 1.7), p < 0.0001), compared with first-time owner surrenders. These results are consistent with those of an Australian study, which showed that among dogs relinquished to a shelter, those originally acquired from a shelter had a lower rate of euthanasia than those from other sources [36].

The second unexpected finding was that over one-third (36.9%) of dogs originally brought in by their owners for euthanasia could, upon further evaluation by staff and discussion with owners, be made available for adoption (with nearly three-quarters of those (71%) placed), for a total save of 21.1% of the original cohort originally brought in to be euthanized. Assessments at intake revealed that some dogs being relinquished for euthanasia may in fact have had medical or behavioral concerns that were amenable to resolution, as opposed to all having terminal health conditions or intractable behavior problems. It is not known whether owners were unaware of the options available to them for helping their dogs, or if they misjudged the seriousness of any concerns. The only study we are aware of that addressed the issue of reasons for surrendering a pet for euthanasia did not indicate that any of those pets were otherwise evaluated or saved [9]. 

In summary, although some characteristics were associated with significantly reduced odds of live release compared to reference categories, in most cases, the absolute live release rate for dogs with a particular characteristic or combination of characteristics often approached or exceeded 90%. Thus, relative measures of risk such as odds ratios should be interpreted in conjunction with absolute live release data associated with a particular trait, and with the understanding the cumulative impact of risk factors when taken in concert may be much less than implied by the presence of multiple statistically significant individual risk factors. Such comparisons may help to identify dogs that may benefit from some extra effort and should not be interpreted to mean that these traits indicate the dogs are not suitable for rehoming. 

### Limitations

The large sample size of this study provided high power to detect small differences that may have little practical importance (e.g., the difference in live release between male and female dogs), so statistical significance should be evaluated in conjunction with the magnitude of the effect size and 95% confidence interval.

The primary goal of this study was to explore factors associated with live release for the population of dogs admitted for the primary goal of rehoming, and not to calculate a global live release rate for the entire population. In this analysis, those dogs that were admitted to the shelter for owner-requested euthanasia and not immediately euthanized were converted to owner surrenders in the data. However, during the analysis, it was discovered that, until the latter months of 2016, it was common practice for staff to label dogs brought in for surrender who were deemed unadoptable at intake as “Owner-requested euthanasia” rather than an “Owner surrender”. Thus, this may have affected the calculation of live release as some unknown portion of the n = 1529 excluded dogs may have been euthanized because of staff assessment and not specifically because of owner intent. 

Some of the data from any shelter database are likely to be more reliable than others. Variables such as date of intake and outcome, intake type, sex, and color are objective and not prone to any specific, systematic, on-going biases or errors. Age can be obtained from owners, or at least roughly estimated into broad categories based on physical characteristics. We believe the categories used here were distinct enough (e.g., puppies, juveniles, young adults, and older adults) to preclude serious misclassification, and if misclassification occurred, that it would be unlikely to extend beyond one category. Other data, however, may be much more subjective. For example, the effect of estimated adult size on live release should be interpreted with caution. Although the trend was as would be expected based on experience, we did not have a direct measure of size in this study. Instead, size was approximated using published weight ranges for purebred dogs [29], using the primary breed designation (a statistic which itself has significant limitations) entered into the database. Condition at intake was a subjective assessment made by intake staff without specific criteria or policy. However, the categories used were generally quite broad (i.e., no concerns, minor concerns, and major concerns) which we believe minimized misclassification. 

Other studies have reported that various aspects of appearance is an important factor when a person is considering adoption [15,16,17,18,31], and there is evidence this was true in this study as well. Breed is an important contributor to appearance, perhaps in part because it is at least roughly determinant of size and color. Typically, the information in shelter databases is from breed designations by staff, which are often based only on a person’s subjective opinion of how closely that morphology aligns with their understanding of various breeds. Even for owner-surrendered dogs, owner-provided information about breed may be suspect, as they may simply be reiterating what someone else has previously also guessed. It has never been demonstrated that either staff guesses, or owner-provided breed designations consistently reflect actual genetic makeup of the dogs involved. The lack of agreement about breed designations among shelter staff members and between shelter staff and DNA testing [20,21,22,23] is not surprising given the classic work by Scott and Fuller, which demonstrated using known crosses of different purebred dogs that the mixed breed offspring may not resemble either parent or each other [37]. Unfortunately, this practice of labeling in the absence of reliable information about heritage has become institutionalized, in part because software packages designed for both shelters and veterinary practice either allow or mandate entering a breed for each dog. The detrimental effect of labeling has been demonstrated by one study, which showed that having different labels may influence the perceptions and decision of potential adopters, despite dogs having similar physical appearance [24]. However, it was not possible to determine whether label or appearance were more influential in adopters’ decisions. Therefore, we created the variable “blockhead-type” dog to capture a wide variety of dogs who may have been considered stigmatized breeds.

Staff and the public may also make assumptions about a dog’s behavioral tendencies, positive or negative, based on breed designations, as well as upon their actual behavior in the shelter. Behavior in the kennel or during interactions with adopters has also been identified as an important factor in adoption decisions [38,39], and it has also been noted that people may generalize bad behavior to other similar-looking dogs [40]. However, detailed information about dog behavior was not available in this database.

With respect to the foster program, it is not possible to determine whether the dogs who were sent to temporary foster homes would eventually have been adopted had they simply remained in the shelter. However, to some degree, this is a moot point, as high-volume shelters such as PACC do not have the space or animal care capacity to permit extensive rehabilitation of dogs who are not good candidates for immediate adoption. Thus, without a suitable alternative, dogs with those special needs must often be euthanized to make space for incoming dogs. In addition, it should be noted that the foster program was not an intervention that was studied prospectively and was analyzed only as a characteristic associated with some dogs. 

We were not able to evaluate the effect of sterilization status because the shelter database did not contain information about sterilization status on intake, only final status, and by statute, dogs being released to a permanent home (i.e., adopted) must be sterilized prior to leaving the shelter. As these dogs had to wait for several days for surgery and recovery from surgery before they could be released to their adoptive home, that period is also reflected in the length of stay calculations. 

The majority of records in this study represent a single intake for a single dog, however, there were 4754 (22.2%) records representing 2191 dogs admitted more than once. It was not unexpected that a minority of dogs would have more complex interactions with a shelter, e.g., coming in as a stray, spending time in foster care, being adopted, and then running at large again and being returned to new owner. We included these records in the analysis, as there would have been no logical reason for selecting either the first, second, or, rarely, third (or higher) intake and outcome as the one to include. This also allowed us to analyze the important effect of being involved in foster care or being returned from adoption, which necessarily involve at least two intakes and outcomes. However, we do not believe this introduced any substantive bias, as the odds ratios for the various other characteristics (excepting these two intake types) did not change substantively when only records for dogs with a single intake or only the last intake for all dogs (n = 18,846) were analyzed (data not shown). 

## 5. Conclusions

These results from two years of data demonstrated it is possible to save the lives of the majority of dogs eligible for rehoming in a large, municipal, open-admission shelter. Furthermore, it is found that some dogs initially brought by their owners to PACC for euthanasia could in fact be rehomed. The foster program at PACC improves the odds of live release, especially for adult dogs. Dogs returned from adoption have a live-release advantage as well, which suggests that the temporary experience in a home is not detrimental and may have facilitated the likelihood of live release after return, akin to a foster situation. Although these results are encouraging, it is not possible to infer establish causal relationships from associations identified in a cross-sectional study, and the foster program is examined only as an intake type, not as a formal intervention studied prospectively. It remains to be determined whether similar results are achievable by a much broader group of animal shelters in the US.

## Figures and Tables

**Table 1:** Disposition of canine intake records downloaded from the Pima Animal Care Center database, 1 January 2015–31 December 2016.

Record Description	n (%)
Total downloaded dog intake records	29,213 (100)
Records excluded, by reason:	7804 (26.7)
Cancelled transactions or missing outcome data	319 (1.1)
Deceased dogs brought for disposal	3323 (11.4)
Dogs not admitted for rehoming or length of stay outside of shelter’s control:	
Rabies quarantine	783 (2.7)
Law enforcement confiscation	1850 (6.3)
Dogs surrendered by owner for euthanasia and euthanized:	
Illness or injury	731 (2.5)
Advanced age	385 (1.3)
Behavior	239 (0.82)
Other/unknown	174 (0.6)
Total records analyzed	21,409 (73.3)

**Table 2:** Outcomes for 21,409 dog intakes at Pima Animal Care Center from 1 January 2015 to 31 December 2016.

Outcome	Number of Intakes	Percent
Adoption	12,651	59.1
Return to owner	2721	12.7
Sent to foster care	704	3.3
Transferred to rescue/shelter partner	2882	13.5
Total live outcomes	18,958	88.6
Euthanized	2275	10.6
Died in shelter or foster care	176	0.8
Total dead	2451	11.4

**Table 3:** Outcomes and median (mean) length of stay for dog intakes at Pima Animal Care Center, for four major categories of intake.

		Live Release		Length of Stay (Days)
Intake Category	Total Dogs	n	Percent	Median (Mean)
Owner surrender	6972	6174	88.6	5 (10)
Stray *	12,777	11,165	87.4	6 (10)
Adoption return	816	791	96.9	4 (10)
Return from foster care	844	828	98.1	0 (6)
Total dogs	21,409	18,958	88.6	

* Length of stay included the mandated minimum holding period required by statute before dogs could be made available for adoption.

## Supplementary Materials

The following are available online at http://www.mdpi.com/2076-2615/8/4/45/s1. Table S1: Odds of live release and odds of live release within seven days for dog intakes at the Pima Animal Care Center, according to characteristics of the dogs.

## Appendix A

Description of the foster care program at the Pima Animal Care Center (PACC) in Tucson, Pima County, Arizona

PACC used a foster care program to provide temporary, interim care in a private home for dogs who were not immediate candidates for adoption due to being below the age limit for placement (eight weeks) or having a treatable health problem and/or manageable behavior problem that was best be addressed outside of the shelter environment. Reasons for this included the following:

- Safety (e.g., a young puppy who is not fully immunized or dogs recuperating from illnesses who would be at risk of many infectious disease if exposed to the general population of dogs in the shelter); and
- Need for regular daily medication or other veterinary treatment that would be impractical in a shelter setting due to the following reasons:
  - The complexity or frequency of the treatment required;
  - The amount of time required for treatment—treatment being required for an unknown duration of time;
  - Better chances of improvement out of the stress of a crowded shelter; and
  - Because intake pressure from new dogs would preclude taking up kennel space for an extended period of time for treatment when a dog was not yet ready for adoption.

The foster program was implemented in 2015. It was supported by an informal network of caregivers in the community who opened their homes to providing temporary care for dogs identified by staff as in need of care outside of the shelter environment. These caregivers were recruited by the shelter through social media, mainstream media (typically via a press release), the organization’s website, and outreach events. Foster homes were not heavily screened by shelter staff (other than to ensure basic safety of all animals), but instead interested people were educated about the dog’s needs and type of care to be provided.

Foster caregivers did not receive any compensation for their participation in the program, nor were they reimbursed for routine care expenses such as food. Participants were provided supplies such as formula for nursing puppies whenever possible. Veterinary care was provided by the shelter veterinary staff.

Foster caregivers were not required to care for any specific number of dogs each year. Typically, an individual caregiver would only take a single dog at a time. The duration of care was determined by the needs of each individual dog, as assessed by shelter staff.

Once a dog is identified as a candidate for foster care, an appropriate caregiver was identified. Persons wishing to participate in the foster program first participated in an orientation and then indicated what type of foster dog they would be interested in accepting. A list of eligible dogs was maintained by shelter staff and updated daily. The foster coordinators would then either cold-call potential matches from the list or send an email with pictures and a description of the dog’s requirements. Once suitable candidate(s) were identified, a “matchmaking” type of conversation, similar to what might happen for an adoption, was conducted to be sure the person understood the level of care required and was able to fully commit to that care. Staff communicated as needed with each foster home to assess the progress of each dog with the caregiver, and then shelter staff determined when the dog may be re-admitted to the shelter for adoption.
