# Supplementary material for: Factors Associated with High Live Release for Dogs at a Large, Open-Admission, Municipal Shelter
Source: Animals (Basel). 2018 Mar 28;8(4):45. doi: 10.3390/ani8040045 (PMC5946129; doi:10.3390/ani8040045)
Supplement: Supplementary file 1 [file animals-08-00045-s001.pdf]

Table S1. Odds of live release and odds of live release within seven days for dog intakes at the Pima Animal Care Center according to the characteristics of the dogs.

	Odds of Live Release (n = 18,958 Live Release/21,409 Dogs)						Odds of LOS [g] ≤7 Days After Intake (n = 12,651 Adopted to Public/18,958 Live Release Dogs)					
	Univariate Logistic Regression			Multivariate Logistic Regression			Univariate Logistic Regression			Multivariate Logistic Regression		
	OR	95% CI	p value	OR	95% CI	p value	OR	95% CI	p value	OR	95% CI	p value
Age category												
Puppy [a] (≤6 months)	1	n/a		1	n/a		1	n/a		1	n/a	
Juvenile (6 –12 months)	0.95	0.81; 1.12	0.57	0.72	0.6; 0.86	<0.001	0.48	0.44; 0.53	<0.001	0.48	0.44; 0.53	<0.001
Young adult (>1 year to less than 3 yrs)	0.78	0.67; 0.9	<0.001	0.66	0.56; 0.77	<0.001	0.35	0.32; 0.38	<0.001	0.36	0.33; 0.39	<0.001
Adult (3 yrs to less than 8 years)	0.7	0.61; 0.81	<0.001	0.55	0.47; 0.65	<0.001	0.38	0.35; 0.42	<0.001	0.35	0.32; 0.39	<0.001
Older adult (≥8 years)	0.47	0.39; 0.57	<0.001	0.36	0.29; 0.45	<0.001	0.45	0.40; 0.52	<0.001	0.39	0.33; 0.45	<0.001
Sex												
Female [a]	1	n/a		1	n/a		1	n/a		1	n/a	
Male	0.73	0.66; 0.79	<0.001	0.68	0.61; 0.76	<0.001	0.96	0.91; 1.02	0.17	0.98	0.92; 1.04	0.46
In type												
Owner surrender [a]	1	n/a		1	n/a		1	n/a		1	n/a	
Stray	0.9	0.82; 0.98	0.016	1.25	1.11; 1.39	<0.001	0.95	0.9; 1.01	0.09	0.98	0.91; 1.05	0.51
Stray [b]	0.73	0.67; 0.8	<0.001	1.01	0.9; 1.13	0.83	n/a	n/a		n/a	n/a	
Return from adoption	4.09	2.73; 6.13	<0.001	4.74	3.02; 7.44	<0.001	1.17	1.01; 1.36	0.041	1.44	1.22; 1.7	<0.001
Return from foster	6.69	4.06; 11	<0.001	5.3	3.13; 8.97	<0.001	3.23	2.68; 3.91	<0.001	3.01	2.44; 3.7	<0.001
Return from foster[c]	22.3	5.5; 89.8	<0.001	22.2	5.48; 90.2	<0.001	2.75	2.09; 3.61	<0.001	3.07	2.45; 3.85	<0.001
Condition at intake												

No concerns [a,d]	1	n/a		1	n/a		1	n/a		1	n/a	
Minor concerns [d]	0.4	0.36; 0.45	<0.001	0.44	0.38; 0.5	<0.001	0.82	0.76; 0.89	<0.001	0.76	0.69; 0.84	<0.001
Major concerns [d]	0.11	0.1; 0.12	<0.001	0.13	0.12; 0.15	<0.001	1.17	1.06; 1.3	0.002	1.04	0.92; 1.17	0.54
Color												
Not black [a]	1	n/a		1	n/a		1	n/a		1	n/a	
Black	1	0.91; 1.1	0.98	0.91	0.8; 1.03	0.15	1.03	0.97; 1.1	0.34	0.98	0.91; 1.06	0.58
Estimated size [e]												
Small [a]	1	n/a		1	n/a		1	n/a		1	n/a	
Medium	0.33	0.29; 0.37	<0.001	0.42	0.35; 0.51	<0.001	0.6	0.55; 0.64	<0.001	0.6	0.55; 0.66	<0.001
Large	0.53	0.46; 0.6	<0.001	0.5	0.42; 0.6	<0.001	0.74	0.68; 0.79	<0.001	0.69	0.64; 0.76	<0.001
Giant	0.38	0.3; 0.47	<0.001	0.57	0.42; 0.76	<0.001	0.74	0.63; 0.86	<0.001	0.81	0.68; 0.97	0.025
Number of breeds listed												
Single breed listed	1	n/a		1	n/a		1	n/a		1	n/a	
>1 breed listed	1.16	1.06; 1.26	<0.001	0.96	0.86; 1.08	0.49	0.79	0.75; 0.84	<0.001	0.72	0.67; 0.77	<0.001
'Blockhead'-type appearance [f]												
No [a]	1	n/a		1	n/a		1	n/a		1	n/a	
Yes	0.37	0.34; 0.41	<0.001	0.46	0.41; 0.52	<0.001	0.66	0.62; 0.7	<0.001	0.72	0.67; 0.77	<0.001

OR, odds ratio; 95% CI, 95% confidence interval for odds ratio; LOS, length of stay; n/a, not applicable. [a] reference category for OR; [b] When 2721 owner-reclaimed strays are excluded from analysis; [c] Excluding puppies and juvenile dogs; [d] Concerns include health and behavioral concerns; [e] based on estimated adult weight for primary listed breed: Small, <~35 lbs; Medium, ~35-65 lbs; Large, ~66-85 lbs; Giant, ~>86 lbs; [f] 'Blockhead' type dog is a morphological category that included any dogs with either a primary or secondary breed attribution in the shelter database that could be consistent with the appearance of a stigmatized breed (e.g., American Staffordshire Terrier, American Pit Bull Terrier, Boxer, American Bulldog, Bull Mastiff, Canine Corso, Dogo Argentino, English Bulldog, Mastiff, 'Pit Bull'-type dog, Rottweiler); [g] Length of stay includes time for in-house surgery and recovery for intact dogs prior to release to adoptive home and does not necessarily just reflect time to adoption decision.
